# Health care costs of home care enzyme replacement therapy for patients with lysosomal storage diseases in Germany

**DOI:** 10.1186/s13023-024-03492-4

**Published:** 2024-12-16

**Authors:** Ria Heinrich, Franziska Claus, Tonio Schoenfelder

**Affiliations:** 1 Scientific Institute of Health Economics and Health System Research (WIG2 GmbH), Leipzig, Germany; 2 Chair Health Sciences–Public Health, Technische Universität Dresden, Dresden, Germany

**Keywords:** Lysosomal storage disease, Enzyme replacement therapy, Home care therapy, Home infusion, Pompe disease, Gaucher disease, Fabry disease, Mucopoly-saccharidosis type I, MPS I, Cost analysis, Germany

## Abstract

**Background:**

Lysosomal storage diseases (LSDs) can be treated with intravenous enzyme replacement therapy (ERT). ERT is being administered either in specialized clinics or in the home care setting. Studies indicate that home-based ERT can be considered safe and positively effects patient reported outcomes. Due to the higher level of individual care required by nurses when administering ERT at home, questions arise whether this will result in additional costs for health care systems. Since cost data to the German health care system are currently unavailable, this study aims to assess home care ERT costs of LSD patients.

**Methods:**

The longitudinal study assessed the direct costs of home-based ERT for patients with LSD diseases Fabry, Pompe, Gaucher, and MPS I from 2019 to 2021 on a patient-by-patient basis by evaluating the healthcare documentation. Costs assessed included ERT drug and further administration equipment, time needed to prepare, administer, and post-process the infusion, and travel expenses of nursing staff.

**Results:**

Data from 62 patients was analyzed: of these, 29 (46.8%) with Fabry disease, 19 (30.6%) with Pompe disease, 10 (16.1%) with Gaucher disease, and 4 (6.5%) with MPS I. Patients ranged in age from 3 to 79 (mean 40 years); 42% of patients reported comorbidities (19.4% with hypertension, and 10% with heart disease). Mean total ERT-related costs were €369,047 per year across all patients. Approximately 98.5% of these costs were attributable to the infusion therapy and administration itself (€363,880), and approximately 1.5% were attributable to personnel and travel costs. Patients with Pompe disease incurred the highest average annual costs (€483,907) and patients with Fabry disease the lowest (€264,896). Cost differences among underlying LSDs were due primarily to ERT drug costs; the drug infused for Fabry disease costs about half as much as that for Gaucher or for Pompe disease. Despite MPS I patients requiring about twice as many infusions, significantly lower drug costs for this disease resulted in the second lowest total mean costs of all LSD subtypes analyzed.

**Conclusion:**

As total costs are almost entirely driven by infusion-related material, especially the ERT drug, moving ERT into the home environment is not expected to increase costs.

**Supplementary Information:**

The online version contains supplementary material available at 10.1186/s13023-024-03492-4.

## Background

Lysosomal storage diseases (LSDs) comprise a group of hereditary metabolic disorders characterized by defective enzymes, resulting in accumulated endogenous waste in various organs of the affected patients. Characterized by a chronic, usually progressive course [1] they manifest in different organs with high phenotypic variability [2]. Each of the different LSDs are rare diseases, and their aggregated global birth prevalence is approximately 1:8,000 [3].

Some of these diseases, including Fabry disease, Gaucher disease, Pompe disease, and mucopoly-saccharidosis type I (MPS I), can be treated with weekly or bi-weekly intravenous enzyme replacement therapy (ERT) [1]. While in some countries, such as the Netherlands, England, and the USA, ERT is regularly administered in the home environment [2], patients with the disease in Germany mainly receive ERT in specialized centers or in general practitioner and specialists clinics. Nevertheless, the German legislature (§39 (1) SGB V [4]) stipulates that outpatient treatment, including home care, is preferable to hospital treatment, provided treatment objectives are maintained. Accordingly, health policy discussions increasingly focus on the further developing of outpatient and home care, which is expected to improve patient care and reduce care costs [2]. In several other indications such as cancer, hemophilia, cystic fibrosis, and multiple sclerosis home infusion therapies have already been shown to be safe, effective, feasible, and cost-saving [5–7]. A systematic literature review also showed positive effects on quality of life and underlines patient preference for home infusion across a range of conditions [6].

Only few studies on the quality of home care ERT are available, and cost data are scarce. For example, Beck et al. investigated the transfer of infusion therapy from inpatient to home care in Fabry disease in Germany [2]. The infusions were well tolerated by all patients and considered to be safe. Patients reported significantly improved quality of treatment in the home environment. Likewise, health-related quality of life (HRQoL) improved, as measured by the dimensions of physical role functioning, physical pain, vitality, social functioning, emotional role functioning, and psychological well-being. The study showed that ERT in the home setting improved patient satisfaction. A more recent study by Concolino et al. investigated the impact of home therapy on safety and compliance in Italian patients with Fabry disease, compared to ERT administered in a hospital setting [8]. Due to a low number of adverse events without recourse to hospital, the home therapy infusions were considered safe. In addition, compliance to treatment was 100% in the home environment, whereas more than a fifth of the patients receiving ERT in hospital had a compliance of less than 90%.

None of these studies assessed the costs of home care ERT and ERT treatment costs to the German health care system are currently unavailable. Additionally, most studies focus on Fabry disease, which is the most common among the LSDs, underrepresenting other subtypes, such as Pompe and Gaucher disease, that also represent a significant proportion of LSDs. Therefore, the present study aims to assess home care ERT costs of patients in Germany with Fabry disease, Gaucher disease, Pompe disease, and MPS I. In this study, home care ERT infusions are delegated by the patient treating physician to specially trained nurses and we assessed the reported direct costs of home care ERT.

## Methods

### Study design and population

The study population comprised all patients with Fabry disease, Gaucher disease, Pompe disease, and MPS I who consented to ERT home therapy, and were deemed suitable for ERT home therapy by their physicians. The cost perspective of the German statutory health insurance (SHI) was considered, since the costs of the LSD home care treatment are covered by the patients` health insurance.

In Germany, patients with LSDs are cared for at home by very few larger organizations that provide specialized care and might travel longer distances to care for patients. Occasionally, there are smaller regional organizations that provide ERT at home. Participating patients who have been cared for by two statewide and one regional home therapy specialty care providers were included in the study. Due to the rarity of the disease and care structures requiring far travel to reach patients, clearly defined regions for analyses were not possible.

We assessed health care service use and direct costs associated with home care ERT from quarter Q1 in 2019 through Q2 in 2021. Due to the short study period, discounting of costs was not performed; however, we quarterly updated infusion-related costs needed for ERT infusions using the current Federal Union of German Associations of Pharmacists (ABDA, German: Bundesvereinigung Deutscher Apothekerverbände) database [9] prices.

### Variables

#### Patient characteristics

We assessed age, sex, occupational status, and the underlying LSD for all patients. Comorbidities, such as respiratory diseases, diabetes mellitus, depression, and heart diseases, were also assessed.

#### Health care resource use

The following health care resource use (HCRU) categories related to home care ERT treatment were assessed and included in our analysis:

- ERT drug and all materials for ERT administration (e.g., syringes, cannulas, puncture needles, mouth guards, compresses, alcohol pads, saline solution, fixation bandage, cannula plaster, latex gloves, infusion filter, rinsing system for prefilled syringes, disinfectant, and disposal container)
- travel distance (in kilometers) undertaken by nursing staff to get to the patient (in some cases, airplane was used in addition to the car)
- duration (in minutes) that nursing staff travelled to the patient
- duration (in minutes) of ERT administration, and
- preparation and post-processing time of ERT administration (in minutes).

For the above mentioned HCRU categories data was directly assessed by the home care nurses, using a standardized data input sheet (see additional file 1). HCRU data were collected per patient and per infusion during the study. The data input sheet was developed together with the home care nurses. They were required to document all material used in ERT administration, as well as the amount and size.

### Cost estimate

Individual cost items were determined from the standardized data sheets using the following methods:

####  ERT drug and all materials used to perform the infusion

Infusion-related costs used for ERT administration were calculated using documented HCRU, the respective PZN (Pharmazentralnummer [pharmaceutical registration number]) of the product (e.g., cannula, plasters, desinfectant), and the price per PZN. The PZN is a nationwide standardized identification number in Germany that is used to uniquely identify articles in the pharmaceutical sector, such as medications, medical aids, and other pharmaceutical products. The PZN identifies an article by the manufacturer, designation, and the package size. Using the PZN, products can be distinguished by size, shape, color, form of dosage, and article type. For every PZN costs are clearly assigned. The price per PZN was taken from the ABDA database [9]. Access is possible for specialist groups and research institutes and is subject to a charge.

For prescription products (e.g., drugs), the established pharmacy retail price (PRP) from the ABDA database was used. The PRP denotes the amount a pharmacy can bill the SHI for a drug. Statutory and individual manufacturer discounts of prescription products were not included, because these are subject to temporary changes, or the contract details of individual health insurance funds with pharmaceutical companies are unavailable.

For products that are available without a prescription (i.e., OTC products such as isotonic saline solution), the PRP used was based on the ABDA database, if this existed. If this was not the case (e.g., for flow compresses and plasters), the pharmacy purchase price (PPP) was used, and the statutory value-added tax of 19% was added, thus deriving a theoretical PRP. This assumption does not imply an intention to make a profit on the part of the pharmacist, since only the purchase price and the statutory value-added tax were considered. This conservative approach was deliberately chosen to avoid overestimating the costs.

The price for a product may vary over time. If the price of the product to which the PZN is assigned changes, the price associated with the PZN is automatically updated in the ABDA database. Price changes for PRP or PPP were checked on a quarterly basis and any price changes were considered for the cost estimates of the study. Since cost changes on drug and material costs during the conduction of the study were considered and taken into account for the cost estimates, it was not necessary to adjust resource use for inflation.

#### Home care nursing staff travel distances and costs

Distances travelled by the home care nursing staff to the patients were covered by different means of transport. To record travel costs as comprehensively as possible, flights were also recorded in addition to travel costs by car. Distances travelled by car were recorded in kilometers. These data were documented by the nursing staff and costs were determined by multiplying by a fixed rate per kilometer, set at €0.30 per kilometer. This rate is the standard for pricing the car travel costs per kilometer in Germany [10]. For distances travelled by air travel, the actual flight ticket prices were applied, documented directly by home care nursing staff. If more than one patient was visited per trip, the prices were divided evenly by the number of patients.

#### Personnel expenses

Personnel expenses include time spent by the nursing staff in caring for the patients that was documented on the standardized sheet. This includes pre- and post-infusion preparation times, as well as time spent caring for the patient during the infusion. In addition, time spent traveling to and from the patients` home were included. The time (in minutes) required, were documented by the nursing staff. This information was converted into hours and multiplied by a fixed hourly wage. The wage for a nurse was set at €18.00 per hour, which corresponds to the average wage of a nurse from one of the participating specialized care providers. An employer’s contribution of 20.925% was added to the labor costs. The employer’s share breaks down as follows: pension insurance: 9.3%, health insurance: 7.3%, Statutory accident insurance: 1.6%, unemployment insurance: 1.2%, and nursing care insurance: 1.525%. The personnel costs do not include education costs for the nursing staff, leasing costs for their cars or additional time spent on office work (e.g., organizing appointments, delegation management) because these are no direct costs.

### Data analysis

The patient characteristics are described as means (SD, range) or prevalence (n, %), as appropriate.

All data was documented separately for each quarter throughout the study period. For the analysis of annually occurring costs, four consecutive quarters with complete data sets were aggregated on a patient-by-patient basis. If two or three consecutive quarters had previously been completely recorded for a patient, the values of the missing quarters were replaced by the mean values of the existing quarters (mean value imputation). Patients with only one, or not even one completely documented quarter, were not included in the cost analysis.

The costs are described as means (standard deviation, SD) and medians per year. To assess significant differences by LSD subtype, an ANOVA was conducted on the total mean costs and all cost categories. As an effect measure, Cohen’s f was calculated for the total mean costs [11]. Further, two-sided t-tests were performed to investigate the cost differences between men and women, between children (< 18 years) and adults (≥ 18 years of age), and between patients with and without comorbidities. Significance level was set at 5%. Cost analysis was performed using the statistical software R (version 4.1.2).

## Results

### Characteristics of study population

Cost data were available for 66 patients. For an annual cost of care analysis, cost data from four consecutive quarters were aggregated, and average costs were extrapolated to one year. Patients with less than two quarters of complete data were excluded from the analysis. This affected 4 patients, leaving data from 62 patients available to be included in the cost analysis. Data were most frequently provided for patients with Fabry disease (29 patients), followed by patients with Pompe disease (19 patients), Gaucher disease (10 patients), and MPS I (4 patients).

The study population consists of 32 males and 30 females. The mean age of the population at baseline was approximately 40 years, with the youngest patient being 3 years old, and the oldest patient 79 years (Table 1). About 42% of the study participants reported comorbidities, among which hypertension (19.4%) was the most common. Nearly 10% had heart disease and about 7% reported having a history of stroke, renal dysfunction, and respiratory disease (e.g., asthma or COPD).

**Table 1:** Study population characteristics at baseline

*Total, N*	62
Fabry disease, n (%)	29 (46.8)
Gaucher disease, n (%)	10 (16.1)
Pompe disease, n (%)	19 (30.6)
MPS I, n (%)	4 (6.5)
*Sex*	
Male, n (%)	32 (51.6)
Female, n (%)	30 (48.4)
*Mean age, years (SD, range)*	39.5 (17.15; 3–79)
Fabry disease, mean (range)	43.9 (8–73)
Gaucher disease, mean (range)	35.8 (17–77)
Pompe disease, mean (range)	38.3 (3–79)
MPS I, mean (range)	23.0 (11–39)
*Occupational status*	
Infant, n (%)	1 (1.6)
Student, n (%)	7 (11.3)
Trainee, n (%)	3 (4.8)
Employed, n (%)	31 (50.0)
Self-employed, n (%)	2 (3.2)
Parental leave, n (%)	1 (1.6)
Unemployed, n (%)	6 (9.7)
Retired, n (%)	5 (8.1)
Disability pensioner, n (%)	5 (8.1)
Unable to work, n (%)	1 (1.6)
*Comorbidities, n (%)*	26 (41.9)
Respiratory diseases, n (%)	4 (6.5)
Diabetes mellitus type I or II, n (%)	1 (1.6)
Depression or other psychological suffering, n (%)	2 (3.2)
Hypertension, n (%)	12 (19.4)
Heart diseases, n (%)	6 (9.7)
Stroke, n (%)	4 (6.5)
Renal dysfunction, n (%)	4 (6.5)
Problems of the gastrointestinal tract, n (%)	3 (4.8)

### Number of infusions and infusion duration

The mean number of infusions per year across all LSD subtypes is 27 infusions per patient and it depended on the underlying LSD: patients with Fabry disease and Gaucher disease received an average of approximately 24–25 infusions per year, and patients with Pompe disease approximately 27 infusions per year. Patients with MPS I received by far the most infusions, approximately 47 per year. The difference in average infusions per year among LSD subtype can be explained by the need for weekly infusions for MPS I patients, and bi-weekly infusions for the three other LSD subtypes assessed in this study. One infantile patient with Pompe disease was an exception, and also required weekly infusions (Table 2).

**Table 2:** Average (SD and range) of infusions by LSD subtype, and time used in travel and infusion administration

*Number of infusions per year and patient*	27.0 (6.58; 19–51)
Fabry disease, mean (SD, range)	24.9 (1.73; 19–27)
Gaucher disease, mean (SD, range)	24.3 (2.71; 20–27)
Pompe disease, mean (SD, range)	27.2 (5.66; 24–50)
MPS I, mean (SD, range)	47.3 (5.00; 40–51)
*Preparation time in minutes, mean (SD, range)*	22.5 (10.74; 8–67)
Fabry disease, mean (SD, range)	21.6 (6.86; 10–34)
Gaucher disease, mean (SD, range)	21.3 (12.03; 8–39)
Pompe disease, mean (SD, range)	25.1 (15.01; 12–67)
MPS I, mean (SD, range)	19.5 (7.26; 14–30)
*Infusion time in minutes, mean (SD, range)*	151.9 (74.75; 44–318)
Fabry disease, mean (SD, range)	114.3 (57.61; 44–195)
Gaucher disease, mean (SD, range)	86.7 (24.09; 65–123)
Pompe disease, mean (SD, range)	231.0 (37.59; 121–318)
MPS I, mean (SD, range)	211.0 (18.29; 191–235)
*Post-processing time in minutes, mean (SD, range)*	17.0 (9.07; 3–55)
Fabry disease, mean (SD, range)	16.6 (8.88; 3–39)
Gaucher disease, mean (SD, range)	13.6 (6.80; 5–28)
Pompe disease, mean (SD, range)	19.1 (10.62; 10–55)
MPS I, mean (SD, range)	17.8 (7.48; 12–29)
*Travel time in minutes, mean (SD, range)*	81.1 (39.63; 5–201)
Fabry disease, mean (SD, range)	71.1 (32.58; 5–158)
Gaucher disease, mean (SD, range)	118.2 (47.00; 64–201)
Pompe disease, mean (SD, range)	80.7 (32.27; 28–137)
MPS I, mean (SD, range)	62.1 (55.96; 11–122)
*Travel distance in kilometers, mean (SD, range)*	102.8 (59.66; 0.5–275)
Fabry disease, mean (SD, range)	88.7 (49.54; 0.5–210)
Gaucher disease, mean (SD, range)	147.6 (74.15; 77–275)
Pompe disease, mean (SD, range)	105.0 (51.14; 20–203)
MPS I, mean (SD, range)	82.8 (90.02; 7–190)

The average infusion duration was approximately 152 min per infusion for the overall study population, with infusions taking anywhere from 44 to 318 min. In addition, an average of 23 min was needed in preparation and 17 min following the infusion, for post-processing (preparation ranged from 8 to 67 min and post-processing from 3 to 55 min) (Table 2). The time required varied, depending on the underlying LSD subtype.

### Travel time and distance

One of the study participants reported self-administering the infusion; no assistance from a nurse was necessary and therefore no related travel costs for this patient were incurred, only the cost of infusion-related materials (ERT drug and equipment used to administer and prepare the infusion). On average, nurse travel time was 81 min to get to the patient (range: 5–201 min), covering an average 103 km (range: 0.5–275 km). Airline travel was only used as a means of transportation until Q3 in 2019, and therefore has only a marginal impact on the total costs (Table 2).

### Costs

#### Total costs

**Fig. 1:**
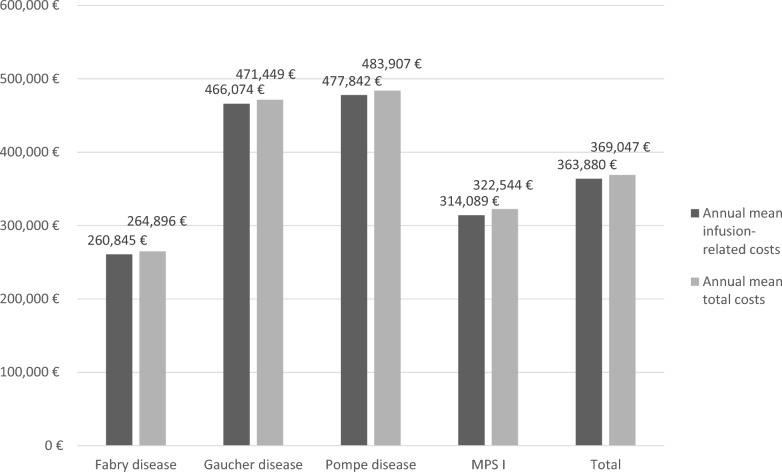
Annual mean infusion-related costs and total costs per patient

ERT-related costs are summarized in Table 3. Mean total ERT-related costs were €369,047 per year, for all LSD subtypes. Most of these total costs (approximately 98%) were attributable to the infusion therapy administration itself (€363,880; e.g., infusion drug, syringes, gloves), as opposed to personnel and travel costs associated with the infusion. The total costs for men (€374,126) were slightly higher than those for women (€363,628) which can probably be attributed to the fact that men are on average heavier than women and therefore require more drug in weight-adapted therapies (such as ERT). Cost differences between men and women were not statistically significant (*p* = 0.7726). Additionally, differences in total costs between children and adults (*p* = 0.0614) and between patients with comorbidities and without comorbidities (*p* = 0.2048) were not statistically significant either. In Fig. 1 the proportion of infusion-related costs compared to the total costs is displayed for all LSDs and the whole population, separately. Per year and patient, the mean personnel costs (specialized nurses) were €3,470, mean travel costs (by car) were €1,641, and mean flight costs were €56. The mean total per infusion costs for all LSD subtypes among all cost categories was €14,141. One infusion for patients with Gaucher disease is, with mean costs of €19,270, most expensive followed by mean total per infusion costs for Pompe disease of €18,326. Fabry patients have mean costs of €10,623 per infusion and patients with MPS I show the lowest mean total per infusion costs of €6,943 but are treated weekly.

**Table 3:** Annual mean total costs and cost categories per patient overall and per indication (in €)

Cost category	All patients (n = 62)	Fabry disease (n = 29)^1^	Gaucher disease (n = 10)	Pompe disease (n = 19)	MPS I (n = 4)	P-Value
Infusion-related costs, mean (SD) [median]	363,880, (142,773.0), [353,515]	260,845, (69,785.9), [260,296]	466,074, (140,393.7), [448,266]	477,842, (117,648.3), [481,786]	314,089, (62,055.6), [329,918]	< 0.0001
Travel costs, mean (SD) [median]s	1,641 (1,026.4), [1,448]	1,356 (774.9), [1,183]	2,198 (1,253.0), [1,688]	1,665 (782.7), [1,641]	2,197 (2,278.5), [2,010]	0.092
Flight costs^2^, mean (SD) [median]	56 (261.6), [0]	62 (233.0), [0]	0 (0), [0]	87 (379.5), [0]	0 (0), [0]	0.827
Personnel costs, mean (SD) [median]	3,470 (1,522.8), [3,227]	2,634 (1,011.5), [2,592]	3,177 (1,222.4), [2,566]	4,313 (1,252.9), [4,214]	6,258 (1,335.3), [5,965]	< 0.0001
Total costs per year, mean (SD) [median]	369,047, (143,204.8), [358,237]	264,896, (69,410.2) [263,931]	471,449, (140,212.9) [452,845]	483,907, (117,383.9) [485,708]	322,544, (64,181.4), [338,444]	< 0.0001, **Cohen‘s f** 1.09

^1^one participant with Fabry disease self-administered the infusion and no travel, flight or personnel costs were incurred

^2^only for two participants with Fabry disease and one participant with Pompe disease the airplane was used

The total costs per year differ significantly (*p* < 0.0001), depending on LSD subtype with an effect size measured by Cohen’s f of 1.09, which indicates a high effect [11] of the LSD subtype on total mean costs (Table 3): patients with Pompe disease incurred the highest average annual costs (€483,907) and patients with Fabry disease the lowest average annual costs (€264,896). These cost differences are due primarily to the difference in the ERT implementation costs (infusion-related costs); the drug infused for Fabry disease costs about half as much as that for Gaucher disease (56%), or the infusion drug for Pompe disease (54%). Even though twice as many infusions are needed in patients with MPS I, the lower drug costs for this disease, in comparison to Gaucher and Pompe disease, result in the second lowest total mean costs of all the LSD subtypes analyzed (Table 3, Fig. 1). For the treatment of Fabry disease and Gaucher disease, there is more than one infusion drug available [1], but the cost differences between the infusion drugs are very small and do not relevantly affect the overall costs.

## Discussion

In this study, we assessed the direct ERT-related costs for a sample of 62 patients with four different LSDs who received home care ERT in Germany. Within the overall study population, we considered 29 patients with Fabry disease, 19 patients with Pompe disease, 10 patients with Gaucher disease, and 4 patients with MPS I.

### Study findings

So far, there are no comparable studies that assess current cost data of ERT home care for LSD patients in Germany. Our cost calculations are based on detailed patient-level data from 2019 to 2021. We found mean total costs of approximately €369,000 per year for LSD patients.

Annual mean total costs differ between LSD subtypes: ERT-treatment of Pompe disease caused the highest annual costs (€483,907) and ERT-treatment of Fabry disease the lowest (€264,896). Cost differences are almost exclusively related to the cost of the infusion drugs used for the ERT. The number of infusions (MPS I), personnel costs, and nurse travel costs, have a very small effect.

About 98.5% of the mean annual total costs were caused by the infusion therapy itself, with the remaining costs only comprising a small proportion of the total amount. There are slight disease-related differences in the proportion of the annual mean total costs incurred by the infusion material (Fabry disease: 98.3%, Gaucher disease: 98.8%, Pompe disease: 98.7%, and MPS I: 97.4%), but this slight difference has no practical relevance for the overall cost consideration.

Personnel expenses (specially trained nurses who administer the home care ERT and their travel costs), accounted on average for about €5,200 or 1.5% of the annual mean total costs. Since infusion drug prices do not differ between ERT home care and ERT at a specialized center or clinic, the proportion of expenses for ERT at home compared to the annual total ERT costs can be considered almost negligible. For LSD care with ERT in a specialized clinic, center or physician’s office, patients would have to visit these institutions regularly, thereby also incurring other direct costs (e.g., travel costs and time, cost of inpatient treatment at the clinic or center) as well as indirect costs (e.g., lost work time for the patient, travel costs and time for family members who accompany the patient). These costs would have to be offset against the travel und personnel costs of the nurses for home care ERT. Whether and to what extent the costs in addition to the infusion drug actually differ between home based ERT and ERT in a specialized clinic, center or physician’s office cannot be answered by the study design applied. This would have required a parallel cost survey for ERT treatment in these institutions. A direct cost comparison, that might also consider lost productivity, patient adherence, and the ability of hospital or center nurses to monitor multiple infusions simultaneously could be taken into consideration for future studies, bearing in mind that the majority of the treatment costs are incurred by the infusion drug itself, independently from the healthcare setting.

ERT home care treatment is often requested by patients and parents, as it is much more convenient and more compatible with everyday life, as it can be better integrated into the patient’s daily routine. Travel costs for patients can be reduced and a burdensome journey to the infusion site can be eliminated, resulting in considerable time savings and reducing the burden on relatives for support. Other findings show that patients perceive home based ERT as an equivalent alternative to ERT in center, clinic, or physician’s office. Additionally, home based ERT can increase quality of life, patient satisfaction, and compliance [1, 8, 12–14]. From a cost perspective, the relatively low additional personnel and travel costs demonstrated in our study may justify ERT therapy at home, since the potential advantages for LSD patients may outweigh the slightly higher expenses to the SHI system, without considering additional costs for the SHI system (e.g., travel expenses, lost work time for patients, costs of inpatient treatment).

### Comparison with other studies

Comparing our results to other studies is difficult since no study on ERT home therapy costs in Germany could be identified. Furthermore, very few articles have been published within the last 10 years, that present cost data on ERT home treatment. Most of these use very small patient samples, cover other LSD subtypes as those included in this study, or mix ERT treatment costs in specialized centers with home therapy costs. Generally, studies from different countries are difficult to compare, because of differences in patient management and in health care reimbursement systems.

Guest et al. modeled the annual costs of treating Fabry patients in Italy [15]. The total costs of ERT with Agalsidase alpha or beta are estimated at around €160,000 per patient and year. In a sensitivity analysis, they showed that home care ERT can even reduce the costs of care slightly compared to treatment at a clinic or center. Compared to the annual costs of about €265,000 per Fabry patient in this study, Guest et al. calculated much lower costs. This may be due to the fact that prices from 2008/2009 were used in their study, that the health care resource use of Fabry disease patients were estimated as average costs per patient by clinical authors and two doctors managing Fabry patients, and that the pricing in the Italian health care system differs from that of the German health care system.

Wyatt et al. conducted a health technology assessment on the effectiveness and cost of ERT in 2011 for different LSD subtypes in the UK [16]. Costs presented include ERT home care and ERT administered in a center, but results do not reveal potential cost differences between both administration methods, so their cost data cannot directly be compared to ours. Wyatt et al. assessed several LSD subtypes and extracted cost data for the four LSD subtypes we evaluated in our study: For Fabry disease, the annual mean ERT costs for adults accounted for £108,242 or £120,840, depending on which infusion drug was considered (Agalsidase alpha 3.5mg, Agalsidase beta 35mg and/or 5mg). The annual cost of care for patients with Fabry disease in addition to the ERT drug costs was estimated at £3,300. The cost of care consisted of hospital costs (e.g., inpatient and outpatient visits, emergency visits, day cases) and services outside the hospital (e.g., GP visits, nursing, therapists, psychologists, etc.). Even though the study design is not directly comparable to ours, the relation of infusion drug costs to the remaining costs are in line with our results; ERT drug costs represent the largest portion of all ERT costs. The portion of costs of care is slightly higher than in our study, but this is due to cost components such as inpatient treatment, which we did not assess. The cost data were also reported for Pompe disease, Gaucher disease, and MPS I. For Pompe disease, the mean annual costs of ERT for adults were £282,798. The annual cost of care for patients with Pompe disease was estimated at £6,300. For Gaucher disease, the mean annual cost of ERT for adults was £126,300 or £144,900, depending on which infusion drug was used (Imiglucerase, Miglustat). The annual cost of care was estimated at £3,000. For MPS I, the mean annual cost of ERT for adults was £258,201 and the annual costs of care were estimated at £8,500 [16].

### Study methodology and limitations

We included a sample of 62 LSD patients receiving ERT at home in our study. The validity of study findings might be limited by the small sample size; however due to the nature of LSDs (the low prevalence of a rare disease), recruiting a large sample is challenging, especially since we only included LSD patients that received their ERT at home by specially trained nurses. Other ERT studies in LSD patients have similar or often smaller sample sizes. Especially when considering LSD subtypes, such as MPS I, the validity of the study findings is limited due to the very low patient number. However, MPS I patients are often multimorbid, placing special demands on ERT home care programs that require certain conditions to be fulfilled, in order for ERT home care to be successfully and safely administered [1]. For this reason, MPS I referrals by physicians to ERT home care may have been low.

Since only patients considered suitable for ERT home care by their physicians were recruited to the study, there is a risk of a selection bias due to the physicians` assessment, which may influence the representativeness of our study findings. Additionally, only patients who had received care at two large and one regional nursing care organization were included in the study. Limiting our patient population to these three providers may have led to a selection of patients; however, the billing of resource consumption for home-based ERT is based on uniform nationwide costs, so it is very unlikely that there is a bias in resource and cost assessment based on region. In terms of age and sex for Gaucher disease, our study sample is comparable to other LSD subtype data: the Gaucher outcome survey (GOS) registry reports a mean age at registry entry of 40.4 years (Gaucher patients in our study 35.8 years) and 44.1% are males [17] (40.0% of Gaucher patients in our study were male). Mean age in the Fabry registry is 36.5 years for men and 40.8 years for women (Fabry patients in our study were an average of 43.9 years old) and the proportion of male patients is 54% [18] (55.2% of Fabry patients in our study were male). Fabry patients in a study conducted by Beck et al. had a median age of 43 years, the proportion of female and male patients were not reported [2].

The cost calculation is based on a detailed resource documentation at the patient level. Nurse documentation errors are possible and may have influenced the calculation accuracy. To ensure the most accurate documentation of resource consumption, standardized forms were used, and the data were checked for plausibility. Since documentation accuracy is relevant to revenue, it is unlikely that resource consumption was under-documented. Cost changes on drug and material costs during the study period were considered for cost calculation. Thus, adjusting resource use for inflation was not necessary since the current resource prices of the SHI were used.

The reported costs in this study only show the direct ERT costs of home care. We did not assess indirect costs and direct costs from other perspectives (such as patient’s or relatives’ ability to work more, by saving long travel times to the center or clinic).

## Conclusion

This study aimed to provide initial data on the direct ERT home care costs for patients with Fabry disease, Gaucher disease, Pompe disease, and MPS I within the German health care context. The total annual costs per patient amount to €369,047 on average across all diseases. There are differences between the LSD subtypes, which can be mainly attributed to the different prices for the types of infusion drugs. The total costs are almost entirely driven by infusion-related costs, in particular by the drug used to prepare the infusion solution. Based on this, moving ERT to the home environment is not expected to increase costs compared to treatment at a center, a clinic, or a physician’s office. Further studies are needed to assess the potential cost difference between home-based ERT and inpatient ERT.

## Supplementary Information

Additional file 1: Data input sheet: The file contains the standardized data input sheet which was used by the home care nurses to document HCRU for each LSD patient.

## Data Availability

The HCRU patient data that support the findings of this study are not openly available because the patient’s declaration of consent specifically states which party (i.e. WIG2) receives the data for further processing. Therefore, the HCRU patient data are available from the corresponding author upon reasonable request. The data used from the ABDA database cannot be made accessible by the authors themselves because access to the data is subject to a charge. However, the data used from the ABDA database can be obtained by interest parties themselves via paid access.
